# Errors Caused by the False Interpretation of the Röntgen Rays, and Their Medico-Legal Aspects

**Published:** 1900-11

**Authors:** Carl Beck

**Affiliations:** New York


### Abstracts and Translations.

### ERRORS CAUSED BY THE FALSE INTERPRETATION OF THE RÖNTGEN RAYS, AND THEIR MEDICO-LEGAL ASPECTS.[1]

BY CARL BECK, M.D., NEW YORK.

SINCE the Röntgen rays began their triumphant march from the modest town on the Main throughout the world, our knowledge of the obscurer ailments has been greatly enlarged, and our methods

[1] Read before the Society of Medical Jurisprudence of New York, May 4, 1900.

of treatment have been revolutionized. It may safely be said that treatises on fractures, for instance, which were written before the Röntgen era, have ceased to be regarded as authoritative. The proofs of the immense usefulness of the Röntgen rays in surgery are so overwhelming, indeed, that to discuss them would be carrying owls to Athens.

Unfortunately, the strangeness of the subject soon attracted many imaginative and speculative minds, that drew unwarranted conclusions and spread erroneous impressions. The ease with which some of the small bones of the human body can be reproduced by the rays on a photographic plate led many medical novices and even ignorant laymen to the indiscriminate use, or rather abuse, of the new discovery. Little wonder that the consequences of such abuse of the rays were soon heralded and misapplied by officious friends, inconsiderate and malicious *confrères,* and last, but not least, by shyster lawyers. It naturally shared the fate of other inventions, as did anæsthesia and asepsis, and as still do many new remedial measures.

The public mind was deplorably disturbed by reports of extensive dermatitis and gangrene of the skin. But while in some individuals a peculiar trophoneurotic idiosyncrasy may exist, predisposing to dermatitis, in the great majority of cases the burns of the skin were caused either by the ignorance of the unskilful operator, the tube often being too near the object, or by too prolonged and too often repeated exposures. Such accidents are not surprising so long as laymen, such as opticians and instrument-makers, who understand nothing of the anatomy and physiology of the skin, are intrusted with "the manufacture of skiagrams." As in many other respects, the question of "proper dosage" must also here be perfectly understood by the operator. A person who irradiates a patient suffering from sycosis, for instance, every day intensely for a whole hour, irrespective of the reaction following such a radical procedure, so that gangrene occurs, is as much qualified to do skiagraphic work as is a shoemaker to prescribe morphine.

Since February, 1896, I have made nearly three thousand skiagraphs, and have never observed the slightest irritation of the skin in any case in which the rays were used for diagnostic purposes. In but two cases did circumscribed depilation supervene. In both skiagraphy of the skull was required frequently and at short intervals. In the first case depilation began after the fifth, and in the second case after the sixth, exposure. Within three weeks the depilated spots had recovered their hair. In addition to such misadventures, crass ignorance is responsible for many distorted Röntgen-ray pictures, which caused such fatal errors that even medical men felt much discouraged by such results. But in considering these errors more closely, it becomes evident that the Röntgen rays never lie, but that it is entirely our own imperfections which induce us to err under peculiar circumstances.

In order to avoid errors, it should in the first place never be forgotten that a so-called Röntgen-ray picture is by no means an ordinary photograph of an object, but a silhouette only (skiagraph),—that is, a photograph of its shadow. To interpret such shadows properly, a thorough knowledge of the normal anatomical relations of the tissues, especially of the bones, that produce such shadows is required. As the most minute gradation of density is registered, it is important to be thoroughly acquainted with the anatomical relations of the bones producing the doubtful shadow. The question, then, would be whether the supposed shadow is normal or not. On certain portions of the skeleton the muscles and tendons would naturally cause obscure shadows. The carpus is especially likely to produce such errors in the skiagraph; the tuberosities of the trapezium, the scaphoid, the hamulus ossis hamati, the os pisiforme, and the eminentiæ carpi volaris, radialis, and ulnaris double up the thickness of the carpus, thereby causing dark shadows, which might be mistaken for foreign bodies. Similar considerations and similar cautions apply to the other diagnostic opportunities offered by the rays.

If a skiagraph of the human hand, for instance, is taken, the plate will show the least light where the bones rest, while the soft tissues appear opaque. There is also a difference of opacity according to the thickness of the tissues, their blood-supply, and their air-capacity. The foot, while easily skiagraphed in the direction of the dorsum towards the planta pedis, from the toes up to the upper third of the metatarsus, presents an obstacle farther backward in the first and third cuneiform bones and the scaphoid, so that it is necessary also to skiagraph the foot on these portions transversely by having the outer face rest on the support. It is by this procedure only that the isolated shadows of the astragalus, the calcaneum, the os cuboidum, the scaphoid, and the fourth and fifth metatarsal bones can be distinctly outlined, so that false interpretations may be excluded. In the early era of the Röntgen rays the normal sesamoids were also sometimes incorrectly interpreted.

How important the knowledge of minute anatomical details is, especially of non-pathological abnormalities, will be evident from the fact that the os intermedium cruris (os trigonum tarsi) has been mistaken for a fragment severed from the astragalus. This bone is a typical part of the tarsus of all mammalia, and its frequency is estimated at from seven to eight per cent. Shepherd, who mistook this bone for a fractured fragment, says, "The fact that this fracture is not mentioned in any of the text-books of surgery or in special treatises on fractures would easily be accounted for by its only being discovered by dissection; it causes no deformity, and the symptoms it would give rise to during life would probably be obscure." The same author tried to produce this fracture artificially on the cadaver, but "in every case," he says, "where this manœuvre was performed, I failed, even when the greatest force was used, to break off the little process of bone mentioned above." Pfitzner regards the os trigonum tarsi as an integral part of the posterior process of the astragalus in the adult, which is analogous to the os intermedium antibrachii.

The significance of a skiagraph for the purpose of estimating the degree of functional disability is not always conclusive. A skiagraph may show a considerable degree of bony deformity after a fracture, and still the function may hardly be disturbed at all. Skiagraphic test has shown that, as a whole, even our best functional results show by no means an ideal union. An unscrupulous patient who secures possession of a skiagraph of his own case, which shows considerable deformity, may, although there is no functional disturbance, strongly appeal to a jury on the strength of his skiagraph, if he succeeds in simulating great impairment. On the other hand, there may be but little evidence of bone injury on the skiagraph, but there may be severe impairment of function on account of the injury to the soft tissues (circulatory, trophic, or inflammatory disturbances), which can be represented only faintly, if at all. This shows the necessity of considering all the other clinical symptoms in connection with the skiagraph.

While it is easy, even for a layman, to understand the significance of most skiagraphs, there are, as alluded to, injuries the correct interpretation of which presupposes, besides thorough anatomical knowledge, the greatest care and a vast amount of experience as to the different modes of delineation in various projection planes. The greatest diagnostic difficulties are offered by the joints. The more complicated a joint is, the more complicated the skiagraphs of its various positions will naturally appear. It is especially the elbow-joint and hip-joint which are kept in view. First of all, the interpretation of the displacement caused by supracondylar fracture of the humerus, and the deformities resulting from it later on, may tax the power of discrimination considerably. The older the fracture, the less conspicuous the fracture line will appear, since it will be more or less overshadowed by the callus. In old fractures the lines cannot be represented as such, and it is only in case of union in a displaced position that its features can be guessed. In the case of a lady aged seventy years, for instance, a second skiagraph, taken three years after a supramalleolar fracture was sustained, showed essentially the same features as the first, which had been taken four weeks after the injury.

In case of the entire absence of displacement, it is only a very distinct skiagraph that shows the line clearly. It is natural that in such cases there is no skiagraphic evidence after recovery,—that is, in from four to ten weeks, according to the type of the fracture. Should a court, for instance, doubt, in such an event, that there had been a fracture, a skiagraph taken after such a period might show a negative result, although there surely was a fracture. In this boy whom I present to you to-night, the very distinct skiagraph, taken only two months after he had sustained a subtrochanteric fracture of the thigh, showed no signs of a fracture. Had this case not been skiagraphed shortly after the injury, no evidence of the fracture could have been subsequently obtained. When no displacement existed, only a faint fracture line will show, but the presence even of a small amount of callus leaves no doubt as to the previous existence of a fracture.

On the other hand, callus formation may be so abundant that, in spite of the absence of displacement, the fullest evidence of fracture may still be furnished after months. In one of my cases, callus formation was so excessive that the attending physician was accused of malpractice, and it was the skiagraph only which convinced the patient that his physician had treated him correctly, the bones being in perfect apposition.

The intra-articular fracture types offer the greatest diagnostic difficulties, inasmuch as the fracture line is also often obscured by the callus formation. If, however, a skiagraph of the other joint is made at the same time, in the same position, and in the same projection, the various delineations of the shadows will be correctly understood and interpreted.

A normal skeleton should also always be compared with the skiagraph. It should particularly be remembered that certain pathological conditions, such as rachitis, for instance, influence the outlines of the bones and may deceptively be supposed to represent a portion of an injury. In such an event the skiagraph of the fellow-extremity will set matters right. In very young children the eminentia capitata appears as if entirely severed from the humerus, although the relations are absolutely normal. The explanation of this very important phenomenon is that the epiphyseal tissues are not sufficiently ossified to produce a shadow on the plate. If these points are not thoroughly considered, a displaced fracture fragment might be erroneously diagnosticated. Union between the epiphysis and the diaphysis of the head of the humerus is not perfect before the twentieth year. The lower epiphysis of the humerus consists of four nuclei, which ossify from the eighth to the seventeenth year. The epiphyses of the trochlea as well as of the olecranon ossify between the seventh and twelfth years, which explains why an osseous nucleus that is still connected with its neighboring epiphyseal nuclei and the diaphysis by cartilaginous tissue appears as an isolated piece of bone which might erroneously be taken for a fragment. The acromio-clavicular junction sometimes shows in the skiagraph a hiatus of the width of a finger, so that a diastasis of the joint might be assumed. But since our knowledge on this new subject has increased, we know that this apparent diastasis is by no means pathological, and that there is a normal gap between the osseous ends of the acromion and the acromial end of the clavicle. The upper epiphysis and the diaphysis of the radius unite between the seventeenth and the eighteenth year, and its lower epiphysis and the diaphysis join in the twentieth year. During the early Röntgen era the translucent space above the epiphyseal cartilage in children was erroneously taken for a fracture line. The head of the femur unites with the diaphysis at the eighteenth or nineteenth year, and the lower epiphysis follows after the twentieth year. The upper epiphysis of the tibia unites with the diaphysis in the twentieth or twenty-second year, while the lower tibial epiphysis unites with the diaphysis between the eighteenth and the nineteenth year.

For the thorough interpretation of skiagraphs in children, it is important to know that at birth the diaphyses of the radius, the ulna, the metacarpal bones, and the phalanges are ossified, while their epiphyses, as well as the whole carpus, are still cartilaginous. It is not before the seventh year that an osseous nucleus shows at the lower epiphysis of the ulna. Union with the diaphysis sometimes begins with the twelfth year, but, as a rule, not before the fifteenth. Even then a small epiphyseal disk remains, which does not disappear before the seventeenth year in the female, and not before the nineteenth year in the male.

In fractures of childhood it should also be remembered that the process of ossification is influenced by various affections of the bone, as, for instance, by rickets.

How important the question of projection is becomes evident when we consider that grave errors may sometimes occur even if all the preliminary conditions required for a thorough understanding of the case seem to be fulfilled. This will appear from the following experience, which has probably not been paralleled in the literature of this subject (compare *New York Medical Journal,* January 6, 1900).

A boy four years of age, while playing in the street, fell against an iron bar. Being unable to rise again, he was taken up and carried to St. Mark's Hospital, where in the first instance moderate pain was noted besides the functional disturbance. There was neither any difference in level or any other deformity, nor any shortening or the typical equinus position. A photograph taken two days after the injury only showed a very moderate and uniform swelling of the leg. Abnormal mobility and crepitus, in accordance, could be produced only by very rough manipulations.

On the day following the injury two skiagraphs were made in different positions, one of them in the dorsal and the other in the lateral position. To my surprise, the one which had been skiagraphed by a direct irradiation, the centre of the platinum disk of the tube being perpendicular to the anterior surface of the leg, did not show the slightest indication of a fracture, while the one which represented the leg irradiated from the outer aspect of the tibia showed a marked fracture line. The fracture presented the typical oblique type in the middle of the tibia, the fracture line running from below anteriorly to above posteriorly, the upper, tapering fragment overlapping the lower end. No sideward displacement having been present, it can be understood why the rays reaching the long axis of the tibia in a vertical direction did not show the fracture line. A very slight change in position, when the inclination towards the fibular direction amounted to less than one millimetre, brought out the fracture distinctly.

Now, if I had, as is the custom in general, taken a skiagraph in the antero-posterior direction only, and if the manipulations made during the first examination were carried out as gently as they properly should be, the fracture might have been overlooked entirely. And if, in view of the local pain and tenderness, the swelling, and the functional disturbance, the possibility of a fracture had been seriously considered, the skiagraph might have silenced the uneasy conscience.

If the case had been brought before a jury, the expert might there on the strength of the first skiagraph have testified in good faith that there was no fracture.

This experience teaches the necessity of adopting the principle of always taking at least two skiagraphs in two different positions in all cases of suspected fracture.

In taking skiagraphs of foreign bodies it must be considered that their size varies according to the distance from the tube. In oblong bodies great errors as to their extent may be committed. Once I was very much surprised in a case in which a needle-fragment had entered the palm of the hand in a perpendicular direction. The plate, while indicating the presence of the needle, distinctly created the impression that the fragment was only about two millimetres in length. When extracted it was found to be more than an inch long, the rays having reached the hand in a perpendicular direction so that the circumference of the fragment was reproduced rather than its length. A side view, of course, would have cleared up the error at once.

Misinterpretations have also arisen from unavoidable mechanical and chemical defects, causing markings in the photographic plate, the significance of which must be well known to the skiagraphic interpreter. Blemishes may also be produced by spots caused by pus from wounds or by perspiration.

In the location of foreign bodies, especially in the skull, many errors were and are still committed. Their avoidance will be considered in a special article.

The case of the German malingerer is a striking illustration of the significance of the Röntgen rays, which for a time proved a protection on account of erroneous interpretation, but showed the case in its true light when assisted by better anatomical knowledge. Soon after the discovery of the Röntgen rays the courts were in a position to grant damages to patients, especially veteran soldiers, who claimed to have been damaged by bullets and were unjustly rejected by medical experts as malingerers. The presence of the bullet, shown on the photographic plate, cannot be denied, and a patient who harbors a piece of cold metal in any part of his body has, as a rule, a good reason to complain. On the other hand, an impostor, who pretends to have been shot and stimulates functional disability, will be exposed by a distinct skiagraph, which would show the absence of the alleged bullet.

It is a question to be solved by my colleagues of the other faculty, whether the court has a right to censure a surgeon for not having used the Röntgen rays in a suitable case, and furthermore whether it can compel a patient in a doubtful bullet—or fracture—case to submit to an exposure to the rays.

A distinct skiagraphic plate will always tell the truth. If accompanied by the registration of the details of operation,—viz., the source of the current (whether battery, static machine, or street), the length of spark of the induction coil, the intensity of the tube, the distance of the platinum disk of the tube from the photographic plate, the position of the object, the kind of plates, and the time of exposure,—it will be a valid document, intelligible to every expert. And together with the anatomical and clinical knowledge of the expert it should be evidence in court.—*Medical Record.*

